# Cardiovascular and musculskeletal co-morbidities in patients with alpha 1 antitrypsin deficiency

**DOI:** 10.1186/1465-9921-11-173

**Published:** 2010-12-07

**Authors:** James M Duckers, Dennis J Shale, Robert A Stockley, Nichola S Gale, Bronwen AJ Evans, John R Cockcroft, Charlotte E Bolton

**Affiliations:** 1 Section of Respiratory Medicine, Wales Heart Research Institute, School of Medicine, Cardiff University, Heath Park, Cardiff. UK; 2 Lung Investigation Unit, Queen Elizabeth Hospital, Birmingham. UK; 3 Child Health, Cardiff University, Heath Park, Cardiff. UK; 4 Wales Heart Research Institute, School of Medicine, Cardiff University, Heath Park, Cardiff. UK; 5 NIHR Nottingham Respiratory Biomedical Research Unit, University of Nottingham, Nottingham City Hospital, Hucknall Road, Nottingham. UK

## Abstract

**Background:**

Determining the presence and extent of co-morbidities is fundamental in assessing patients with chronic respiratory disease, where increased cardiovascular risk, presence of osteoporosis and low muscle mass have been recognised in several disease states. We hypothesised that the systemic consequences are evident in a further group of subjects with COPD due to Alpha-1 Antitrypsin Deficiency (A1ATD), yet are currently under-recognised.

**Methods:**

We studied 19 patients with PiZZ A1ATD COPD and 20 age, sex and smoking matched controls, all subjects free from known cardiovascular disease. They underwent spirometry, haemodynamic measurements including aortic pulse wave velocity (aPWV), an independent predictor or cardiovascular risk, dual energy X-ray absorptiometry to determine body composition and bone mineral density.

**Results:**

The aPWV was greater in patients: 9.9(2.1) m/s than controls: 8.5(1.6) m/s, p = 0.03, despite similar mean arterial pressure (MAP). The strongest predictors of aPWV were age, FEV_1_% predicted and MAP (all p < 0.01). Osteoporosis was present in 8/19 patients (2/20 controls) and was previously unsuspected in 7 patients. The fat free mass and bone mineral density were lower in patients than controls (p < 0.001).

**Conclusions:**

Patients with A1ATD related COPD have increased aortic stiffness suggesting increased risk of cardiovascular disease and evidence of occult musculoskeletal changes, all likely to contribute hugely to overall morbidity and mortality.

## Background

The systemic manifestations in chronic respiratory conditions such as chronic obstructive pulmonary disease (COPD) can be multiple and include a persistent systemic inflammatory state, excess cardiovascular disease, bone thinning and low skeletal muscle mass; each associated with considerable morbidity and mortality[1-3]. Alpha-1 antitrypsin deficiency (A1ATD) is an established genetic risk factor estimated to occur in 1-2% of patients with COPD, though generally under-recognised and the co-morbidities in this condition have not explored [4]. Characterisation of the genetic and proteonomic basis of the A1ATD status has supported the protease-antiprotease theory of lung injury, as abnormally low levels of alpha-1 antitrypsin (A1AT) leads to unopposed protease activity and destruction of the elastin matrix within the lung, resulting in emphysema. In usual COPD, the emphysematous extent has been related to the cardiovascular and bone alterations [5,6].

In addition to its presence in the lungs, circulating A1AT also binds to the endothelium [7], has a protective role in limiting vascular damage and is involved in the regulation of vascular smooth muscle cells and control of inflammatory pathways [8,9]. Low levels of A1AT, leading to unopposed neutrophil elastase activity in the vasculature may result in the local degradation of elastin, with a resultant increased collagen deposition. In the larger central arteries, the elastin:collagen balance is an important structural contribution in determining arterial stiffness, which is implicated in arteriosclerosis and subsequent cardiovascular risk[10,11]. The current literature on cardiovascular risk in the PiZZ A1ATD is conflicting, largely due to small sized studies or sub-analysis of this homozygous state in larger studies [12-14].

Previously, we have demonstrated increased aortic stiffness, low bone mineral density (BMD) and low skeletal muscle mass in the presence of a heightened circulating inflammatory state in patients with usual COPD [15,16]. We hypothesised that these cardiovascular and musculoskeletal systemic co-morbidities would be present in the chronic respiratory disease A1ATD and hence need to be identified.

## Materials and methods

### Study Subjects

Adult patients with PiZZ A1ATD and confirmed airways obstruction or CT proven emphysema on the ADAPT (Antitrypsin Deficiency Assessment and Programme for Treatment) register residing within a 1.5 hour commutable distance of the Cardiff study centre, together with patients from respiratory out-patients clinics were invited. Patients were studied when clinically stable [15]. Sedentary control subjects, matched for age, sex, smoking and additionally free of respiratory disease were also recruited from the same region. Exclusion criteria for all subjects included malignancy, rheumatoid disease, diabetes mellitus, maintenance oral corticosteroids, active infection or other chronic inflammatory disease, weight losing drugs, known congestive heart failure, documented history of cardiovascular disease, solid organ transplantation, parallel participation in interventional study, pregnancy or breastfeeding. All subjects gave written, informed consent and the study had South West Wales Research Ethics Committee approval (08/WMW02/48).

### Anthropometry, Lung Function and Physical function

Height and weight (Seca, Germany) were determined barefoot and in lightweight indoor clothing and the body mass index (BMI) calculated. A low BMI was defined as <20 kg/m^2 ^[15].

All subjects performed spirometry: forced expiratory volume in 1 second (FEV_1_), forced vital capacity (FVC), and FEV_1/_FVC ratio, (Vitalograph Ltd, Bucks UK) having withheld short- and long-acting bronchodilators for six and twelve hours respectively. Subjects underwent a six minute walk test (6MWD) after all other assessments had been performed [17]. All patients completed a St George Respiratory Questionnaire (SGRQ) and all subjects completed a physical activity questionnaire relating to their last month's activity [18,19]. Contemporaneous room air arterialised ear lobe gases were determined in the patients. Recent (within 12 months) post bronchodilator lung volumes and gas transfer data were obtained from patients' medical notes and, if not, were performed (n = 3).

### Cardiovascular measurements

Subjects were studied after an overnight fast and 6 hours after abstinence from caffeine, tobacco, and inhaled short-acting β_2 _agonists and 12 hours following long-acting β_2 _agonists, to ensure haemodynamic measures were made free of the acute effect of these agents [16]. All tests were performed after ten minutes of supine rest. Peripheral blood pressure (BP) was measured in the dominant arm (OMRON Corporation, Japan). Radial artery waveforms were recorded via a high fidelity micro manometer (Millar Instruments, Texas). Pulse wave analysis (Syphgmocor, AtCor Medical, Australia) was subsequently used to derive a heart rate adjusted augmentation index (AIx-75). Aortic (carotid-femoral) pulse wave velocity (aPWV) was measured as previously described [10,16]. Traces were assessed by an independent reviewer, blinded to the subject status.

### Dual-Energy X-ray Absorptiometry (DXA)

Whole body composition and BMD at the lumbar spine and hip (and 3 hip subregions) were determined, within 3 weeks of the main study visit, using Hologic Discovery (Hologic, MA)[15]. The FFM, indicative of skeletal muscle mass was expressed as a height squared ratio to give an index: FFMI. A low FFMI was defined as < lower 5^th ^percentile for controls [15]. Upper limb (both), lower limb (both) and trunk FFMI were calculated using anatomical markers [20]. Osteoporosis was defined as T score less than -2.5 and osteopenia as T score less than -1 but greater than -2.5 [21].

### Biochemistry, bone markers and inflammatory mediators

An early morning, fasted venous blood sample was collected. Testosterone (men only), thyroid function tests (TFT's), parathyroid hormone (PTH) and insulin were measured using immunoassay (ADVIA Centaur). 25-hydroxy (OH)-Vitamin D was measured using direct competitive luminescence (Diasorin Liason). Corrected calcium, creatinine, fasting glucose and lipids (cholesterol, High Density Lipoprotein [HDL], Low Density Lipoprotein [LDL] and triglycerides) were measured using two site immunoassay (Abbot Diagnostics, Berkshire). IL-6 was measured using Quantikine immunoassay (R&D Systems Inc, MN, USA), as previously reported [15].

### Data Analysis

Data analysis was performed using the Statistical Package for the Social Sciences (SPSS, Chicago, IL), version 12.0. Log_10 _transformation was used where data was not normally distributed. Results are presented as arithmetic or geometric mean and standard deviation (SD). Analyses included χ^2 ^test, independent t test, Pearson's correlations, one-way analysis of variance with post hoc Tukey analysis, and stepwise multiple regression analysis. A p < 0.05 was considered significant.

Sample size was based on our primary outcome of aPWV, using data for patients with COPD. 18 patients were required per group in order to detect a 15% difference in aPWV between groups, with a SD of 2 m/s, a significance of 0.05 and a power of 0.8.

## Results

### Subjects

Of the total 49 patients with PiZZ A1ATD approached, 23 agreed to be contacted and fulfilled the initial screen of suitability, but three were subsequently excluded after further discussion: due to known ischaemic heart disease (n = 2) and active Hepatitis C infection (n = 1). Of the 20 patients recruited, one developed chest pains necessitating investigation prior to study commencement and he was excluded. Thus, 19 A1ATD patients and 20 controls were studied.

Patients and controls were similar in terms of age, sex, height and smoking pack year history, (Table 1). Of the patients, four had never smoked and five smoked less than 10 pack years and one was a current smoker. Of the control subjects, three had never smoked and eight smoked less than ten pack years and two were current smokers.

**Table 1:** Subject demographics, pulmonary function and body composition

	Controls n = 20	Patients n = 19	P value
**Age (years)**	61.1 (9.1)	59.2 (12.1)	0.59
**Men n (%)**	13 (65%)	12 (63%)	
Smoking pack year*	5.5 (0-70)	10.0 (0-60)	0.69
**FEV_1 _(% predicted)**	100.8 (12.5)	42.7 (23.3)	< 0.001
**FVC (% predicted)**	106.7 (17.5)	87.2 (25.6)	0.008
**TLCO (mmol/min/kPa)**	ND	4.1 (1.3)	
**Oxygen saturation (%)**	97 (1)	92 (4)	0.008
PaO_2 _(kPa)*	ND	8.9 (2.0)	
**6 MWD (m)**	497 (80)	312(133)	< 0.001
**Height (m)**	1.69 (0.06)	1.71 (0.10)	0.42
**Weight (kg)**	80.5 (14.8)	69.4 (12.0)	0.014
**BMI (kg/m^2^)**	28.3 (4.0)	23.7 (3.0)	0.001
**FFMI (kg/m^2^)**	18.6 (2.6)	15.7 (1.7)	< 0.001
**SGRQ - total**	ND	52.0 (15.2)	
**Physical activity score**	40 (9)	33 (5)	0.006

All data represented as mean (standard deviation) unless otherwise indicated

*median (range)

Abbreviations: **FEV_1 _**Forced Expiratory Volume 1 second, **FVC **Forced Vital Capacity, **TLCO **Total Lung Carbon Monoxide Transfer Factor, **PaO_2 _**Arterial oxygen tension, **6MWD **6 Minute Walk Distance, **BMI **Body mass Index, **FFMI **Fat Free Mass Index, **SGRQ **St Georges Respiratory Questionnaire

**ND **not determined

Of the patients, 17 were prescribed combination long acting β_2 _agonist/inhaled corticosteroid (median steroid dose 2000 mcg betamethasone equivalent). Inhaled anticholinergics were prescribed to 16 patients. Three patients had a prior diagnosis of osteoporosis and were receiving bisphosphonate therapy. There were four patients and three controls with a prior diagnosis of hypertension. Three patients and three controls had a prior history of hypercholesterolaemia. The median (range) alpha 1 antitrypsin levels in patients was 3.1 (1.7-8.6)μmol/L

### Pulmonary and physical function tests

As expected, the patients had a lower FEV_1_, FVC and a shorter 6MWD than the controls, (p < 0.001), (Table 1). Two patients had a resting PaO_2 _on room air <7.3 kPa.

### Cardiovascular data

There was no difference in heart rate, peripheral or central systolic, diastolic BP or mean arterial pressure (MAP) between patients and controls, Table 2. Mean (SD) aPWV was greater in patients: 9.9(2.2)m/s than controls: 8.5(1.6)m/s, p = 0.03). AIx-75 was not different, p = 0.33, (Table 2). The aPWV was greater in males than females overall, p < 0.001 as well as in each group. We found increased aPWV in patients with A1ATD compared to controls by at least 0.7 m/s (and upto 1.8 m/s) for each decade in the overlapping age range, however small numbers per group preclude formal analysis to determine significance. In all subjects, aPWV was associated with age (r = 0.56, p < 0.001) and Log_10_IL-6 (r = 0.50, p = 0.001) and was inversely related to FEV_1_% predicted (r = -0.43, p = 0.006), FVC% predicted (r = -0.42, p = 0.007) and physical activity scores (r = -0.36, p = 0.02).

**Table 2:** Subject haemodynamics

	Control n = 20	Patient n = 19	P value
**Heart rate (bpm)**	68 (12)	76 (13)	0.07
**Peripheral systolic BP (mmHg)**	136.9 (14.9)	130.3 (14.3)	0.17
**Peripheral diastolic BP (mmHg)**	82.7 (6.9)	84.6 (7.5)	0.40
**Peripheral MAP (mmHg)**	100.4 (10.2)	101.5 (9.2)	0.72
**Peripheral PP (mmHg)**	54.2 (16.6)	45.7 (12.0)	0.08
**Central Systolic** **pressure (mmHg)**	126.5 (14.5)	121.1 (14.5)	0.26
**Central diastolic** **pressure (mmHg)**	84.3 (7.1)	85.8 (7.5)	0.53
**Central MAP (mmHg)**	102.0 (7.5)	101.5 (9.2)	0.86
**Central PP (mmHg)**	43.0 (15.6)	35.2 (10.8)	0.08
**aPWV (m/s)**	8.5 (1.6)	9.9 (2.1)	0.03
**AIx-75**	23.7 (8.8)	26.1 (6.5)	0.33

Abbreviations:

**MAP **Mean arterial pressure, **PP **pulse pressure, **aPWV **aortic pulse wave velocity, **AIx-75 **Augmentation index corrected to heart rate 75 beats per minute

Stepwise linear regression in all subjects with aPWV as the dependent variable and age, sex, FEV_1_% predicted, peripheral MAP, heart rate, log_10_IL-6 and smoking pack years inputted as independent variables, demonstrated that the predictors were age (R^2 ^= 0.30, p < 0.001), FEV_1_% predicted (R^2 ^= 0.23, p = 0.001), peripheral MAP (R^2 ^= 0.08, p < 0.001) and heart rate (R^2 ^= 0.09, p = 0.002).

### Body composition

The mean BMI was lower in patients than controls (p = 0.001) (Table 1), and 2 patients had a pathologically low BMI (no controls). The 2 patients with a low BMI also had low FFMI and 6 other patients had a low FFMI with normal BMI, indicating hidden FFM loss. The FFMI was globally lower including upper limbs (p = 0.01), lower limbs (p < 0.001) and trunk (p = 0.001) of patients compared to controls. In the whole study population, FFMI was related to FEV_1_%predicted (r = 0.40, p = 0.01) and 6MWD (r = 0.46, p = 0.003).

### Bone mineral density and osteoporosis

The BMD at the total hip site, all three hip sub-regions and the lumbar spine were all less in the patients than controls (p < 0.001), (Table 3). In all subjects, FEV_1_% predicted was related to total hip BMD (r = 0.60, p < 0.001) and lumbar spine (r = 0.55, p < 0.001). Similarly, FFMI was related to the total hip BMD (r = 0.67, p < 0.001) and lumbar BMD (r = 0.61, p < 0.001).

**Table 3:** Subject bone mineral density and profile

	Control n = 20	Patient n = 19	P value
**BMD Hip (g/cm^2^)**	1.03 (0.20)	0.79 (0.12)	<0.001
**BMD Lumbar spine (g/cm^2^)**	1.12 (0.20)	0.88 (0.13)	<0.001
**PTH (pmol/l)**	5.26 (1.88)	5.53 (2.00)	0.67
**25-OH Vitamin D (μg/l)**	18.0 (8.6)	19.5 (9.0)	0.62

All data represented as mean (standard deviation) unless otherwise indicated

# geometric mean

Abbreviations

**BMD **Bone Mineral Density, **PTH **parathyroid hormone **25-OH **25 hydroxy

Multiple regression analyses with total BMD at either the lumbar spine or hip as the dependent variables and age, sex, FEV_1_% predicted, FFMI as independent variables were performed. At both the hip and the lumbar spine, FFMI, (adjusted R^2 ^= 0.43, p < 0.001 and R^2 ^= 0.35, p < 0.001 respectively) was the only predictive variable.

There were eight patients and two controls with a clinical diagnosis of osteoporosis at either site. Seven of the eight patients and both the controls were new diagnoses of osteoporosis and not previously clinically suspected, necessitating initiation of therapy. Two further patients on prior bisphosphonate therapy were no longer classified as osteoporotic on DXA scan, although still osteopenic. In total, there were 8 A1ATD patients and 4 controls who were osteopenic. There was no difference in FFMI, FEV_1_% predicted, 6MWD and aPWV between those patients with osteoporosis or not.

### Biochemistry and systemic inflammation

In order to determine other factors that might influence osteoporosis, we explored TFT's, testosterone, 25-OH vitamin D and PTH levels. No subject had abnormal TFT's and no male subject had low early morning testosterone levels (< 8.0 nmol/L), (Table 4).

**Table 4:** Biochemistry and Systemic Inflammatory status

	Control n = 20	Patient n = 19	P value
**Total cholesterol (mmol/l)**	5.8 (1.0)	5.7 (1.4)	0.66
**HDL (mmol/l)**	1.6 (0.3)	1.7(0.5)	0.44
**LDL (mmol/l)**	3.7 (1.0)	3.5 (1.2)	0.58
**Triglycerides # (mmol/l)**	1.19 (0.49)	0.98 (0.31)	0.12
**Fasting Glucose (mmol/l)**	5.2 (0.6)	4.9 (0.5)	0.06
**Creatinine (μmol/l)**	88.4 (18.1)	84.0 (12.6)	0.39
**eGFR (mls/min/1.73 m^2^)**	75.9 (15.6)	79.4 (12.1)	0.44
**Testosterone(males)nmol/l #**	15.6 (1.2)	24.6 (1.4)	0.001
**IL-6 (pg/ml)#**	2.18 (1.62)	3.24 (1.62)	0.016

All data represented as mean (standard deviation) unless otherwise indicated,

# geometric mean

Abbreviations: **HDL **High Density Lipoprotein, **LDL **Low density Lipoprotein, **eGFR **Estimated Glomerular Filtration Rate; **IL-6 **Interleukin 6

Insufficient 25-OH vitamin D levels (< 20 μg/l) (21) were recorded in 12 patients and 12 controls. Of these, three patients and two controls had low 25-OH vitamin D levels (< 10 μg/l), (Table 3)[22].

Elevated PTH levels (all with normal calcium and creatinine levels) were recorded in 9 patients and 10 of the controls. Of these, elevated PTH levels in conjunction with low 25-OH Vitamin D levels but normal calcium levels were seen in five patients and eight controls.

All patients with A1ATD had normal liver function tests. There were 7 patients and 7 controls with total fasting cholesterol >6.2 mmol/l, and one patient had an impaired fasting glucose of > 6.0 mmol/l. Circulating IL-6 was greater in patients: (3.24(1.62) than controls: 2.18(1.62) pg/ml, p = 0.016).

## Discussion

This study demonstrates, for the first time, that patients with COPD due to alpha 1 antitrypsin deficiency have increased aortic stiffness, as determined by aPWV, compared to age and sex matched controls despite a similar risk profile including blood pressure, fasting lipids, glucose and a comparatively low smoking pack year exposure. This indicates increased cardiovascular risk in this group of subjects with A1ATD with established chronic lung disease that would not have been detected on standard peripheral sphygmomanometry. In addition, patients with A1ATD had lower BMD than matched controls with a significant proportion having a new diagnosis of osteoporosis despite none receiving maintenance oral corticosteroids. Patients had lower BMI and FFMI than controls with nearly half the patient group having skeletal muscle mass loss [15].

The study has highlighted a number of potential mechanisms for these changes. As in usual COPD where systemic manifestations have been explored in more detail, it is possible that the heightened systemic inflammatory state, physical inactivity and impaired muscle function may have a role in the development of the systemic manifestations [15,16]. However, in addition, the systemic protease-antiprotease imbalance, at the core of A1ATD, is a possible central factor.

The increased systemic inflammatory state in the patients, when clinically stable and heightened at times of exacerbation may be a mechanism of the altered large artery haemodynamics [15,23]. This relationship corresponds to the literature between systemic inflammation and cardiovascular risk in both the general population and in patients with COPD [16,24]. Inflammation is associated with endothelial dysfunction but may also promote arterial stiffness by direct effects on vessel architecture and vascular smooth muscle function [25-27]. The association of A1AT with inflammatory and coagulation pathways and the A1AT link to HDL and its functional properties are interesting considerations suggesting a pleiotropic beneficial effect of A1AT, which the deficient state exposes [8,9,28].

Importantly, the A1AT deficiency may have a direct effect on the elastin of the larger arteries, leading to altered structure and function. Talmud *et al*. described a protective role for A1AT in the progression of established coronary artery atherogenesis, based on angiography and the genotyping of 800 subjects in 2 clinical trials [13]. Indeed, Aldonyte *et *al. demonstrated that A1AT is taken up by pulmonary endothelial cells using *in vitro *porcine cultures and appears to have a protective role against cigarette smoke induced endothelial apoptosis [29]. Similarly, the transformational polymerisation of AAT in A1ATD subjects, present not just in hepatocytes but circulating and in the lung and systemic endothelium are pro-inflammatory and inactivate the protein as an inhibitor of proteases (thereby reducing any anti-protease protection further) and hence may be contributory to the altered haemodynamics [30,31]. Indeed, such a protease - antiprotease imbalance mechanism may account for the cross-sectional association of emphysema severity (using CT) with both low BMD and increased arterial stiffness in usual COPD [5,6]. However, in these COPD studies, knowledge and quantification of protease - antiprotease activity and level are not described. Interestingly, McAllister *et. al*. reported that emphysema severity was an independent variable, over airways obstruction and the systemic inflammatory mediator CRP, for brachial PWV in patients with usual COPD [5]. It is conceivable that a systemic proteolytic process is occurring in the emphysematous spectrum of COPD including A1ATD subjects affecting arterial wall structure; however conclusive proof either way is beyond the scope of this study.

Increased aortic distensibilty, a suboptimal measure of arterial stiffness, has been reported in a small number of male patients with A1ATD but not the female subset where the authors sought to assess the risk of aortic aneurysms [12]. The use of historical controls and the wide age range from early adulthood limits its interpretation. Meanwhile, other reports have postulated that A1ATD may actually be associated with a reduced risk of ischaemic heart disease and lower BP, however subject numbers were low, especially of the homozygous PiZZ genotype, n = 6 [32].

Increased arterial stiffness, measured by aPWV confers an increased and independent cardiovascular risk [10,11]. This has been firmly established through a wealth of studies in both general populations and various at-risk disease groups [8]. Addressing central haemodynamic measurements have become key outcome measures in cardiovascular studies, as alterations in large artery haemodynamics may not be detected using only standard peripheral measurements [33,34]. The clinical relevance of this increased cardiovascular risk is demonstrated by the high cardiovascular mortality seen in COPD patients in general [3,35]. Patients with A1ATD often present at a younger age than usual COPD and indeed, here, the subjects were about 5 years younger than subjects in previously reported usual COPD studies, which have previously excluded A1ATD. Despite this younger age group, the patients with A1ATD have striking systemic consequences and may well reflect an accelerated ageing process [36,37]. The extent of these systemic consequences in usual COPD has only recently been prioritised and we now highlight in A1ATD. Optimisation of risk factors is imperative and exercise regimes or pulmonary rehabilitation may offer additional improvement in any functional alteration leading to arterial stiffness [38]. The similar results for AIx-75 between the 2 groups are in keeping with the non-linear relationship with age of this measure, which plateaus over the age of 50 years and hence deemed less sensitive to change in this age group.

The mechanism of low BMD in these patients is similarly likely to be due to several mechanisms. Some patients had been exposed to short courses of oral corticosteroids although none were on maintenance therapy. Previously, we have reported a lower BMD occurs in patients with usual COPD even if steroid naïve suggesting other "COPD related factors" are likely to be additional key factors [39]. In this current study, Vitamin D levels were low across both patients and controls suggesting it may play only a limited or no additional role. To allow for seasonal variation in Vitamin D, all subjects were studied during winter period. Like cardiovascular disease, a low BMD may be related to the circulating inflammation but additionally to deconditioning as evidenced by the low FFM, the shorter 6MWD and lower physical activity scores seen in these patients compared to controls. Interestingly, however, the FFMI was globally low throughout all the body regions and not confined to lower limbs. Previously we have reported that patients with usual COPD with osteoporosis had elevated aPWV [16], however the current study was not powered for such an outcome and description of the co-morbidities was our primary aim.

### Limitations of study

The study size is small due to difficulty in recruiting eligible Pi ZZ A1ATD patients within a commutable distance of the research centre. The comprehensive exclusion criteria prevented other confounders, such as diabetes or known cardiovascular disease from exaggerating or affecting the primary aim of identifying the hidden co-morbidities. Collaboration with ADAPT ensured the sample size was sufficient to achieve our aims based on the power calculation. A proportion were ex or current smokers, albeit more modest exposure than usual COPD patients. This is inevitable given smoking remains the trigger that precipitates the emphysematous change in A1ATD and hence presentation to physicians. Emphysema index was not measured universally in subjects and hence not included.

The cross sectional nature of the study does not allow causal relationships between A1ATD, arterial stiffness, low BMD and systemic inflammation to be inferred but does show relationships that would suggest that further longitudinal studies may be informative and highlights a priority for full assessment of the co-morbidities in patients with A1ATD. Lastly, the presence of such extrapulmonary manifestations in patients with the heterozygote state remains to be explored and may indicate whether the presence of the Z protein alone plays a role rather than the low A1AT level.

In conclusion, patients with COPD related to PiZZ A1ATD had increased aortic stiffness compared with age and gender matched controls without chronic lung disease, despite the similar risk profiles such as smoking, blood pressure and lipid levels. Such patients also had a lower BMD with evidence of osteoporosis and a low skeletal muscle mass. There is thus a priority to explore underlying mechanisms as to why patients COPD due to A1ATD are at risk of these systemic consequences, which are hugely under-recognised in clinical practice but contribute to morbidity and necessitate optimal management.

## Funding

Dr James Duckers was supported by a Cardiff and Vale NHS Trust Clinical Research Fellowship. Dr Charlotte Bolton is funded by NIHR Nottingham Respiratory Biomedical Research Unit. ADAPT (Antitrypsin Deficiency Assessment and programme for Treatment) is funded by an unrestricted grant support from Talecris.

## Competing interests

JD has no conflicts of interest to disclose. DJS has no conflicts of interest to disclose. RS has no conflicts of interest to disclose. Talecris funds the ADAPT programme in Birmingham of A1ATD subjects. However, ADAPT was one of several sources of subjects; Talecris had no involvement in the funding, the design, analysis or interpretation of this study; NSG has no conflicts of interest to disclose. BAJE has no conflicts of interest to disclose. JRC has no conflicts of interest to disclose. CEB has no conflicts of interest to disclose.

## Authors' contributions

JD: helped design the study, conducted the clinical assessments, analysed and interpreted data and wrote the first draft; DJS: helped design the study and contributed to the interpretation and writing; RS: helped design the study and contributed to the interpretation and writing; NSG: assisted with the conduct the study and contributed to the writing; BAJE: helped design the study and contributed to the writing; JRC: helped design the study and contributed to the writing; CEB: helped design the study, assisted with conduct of study, analysis and interpretation and helped write the manuscript. She is responsible for the integrity of the data analysis. All authors have read and approved the final manuscript.
